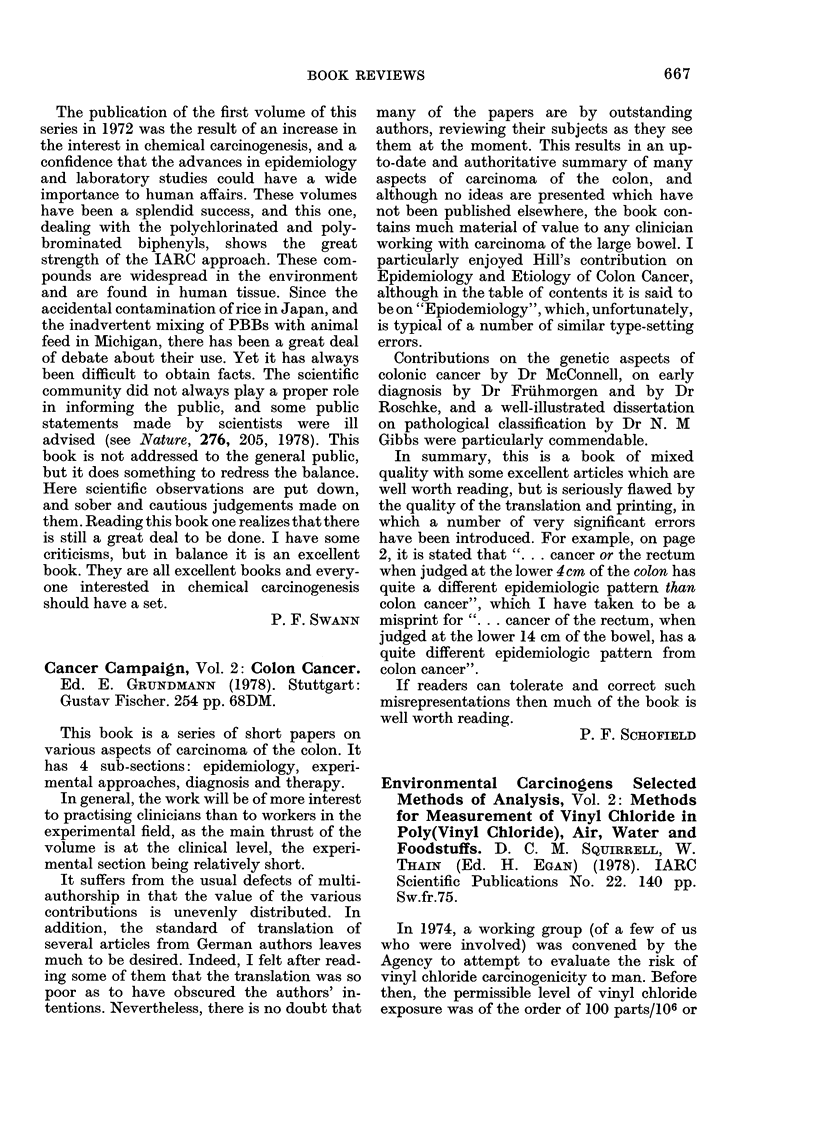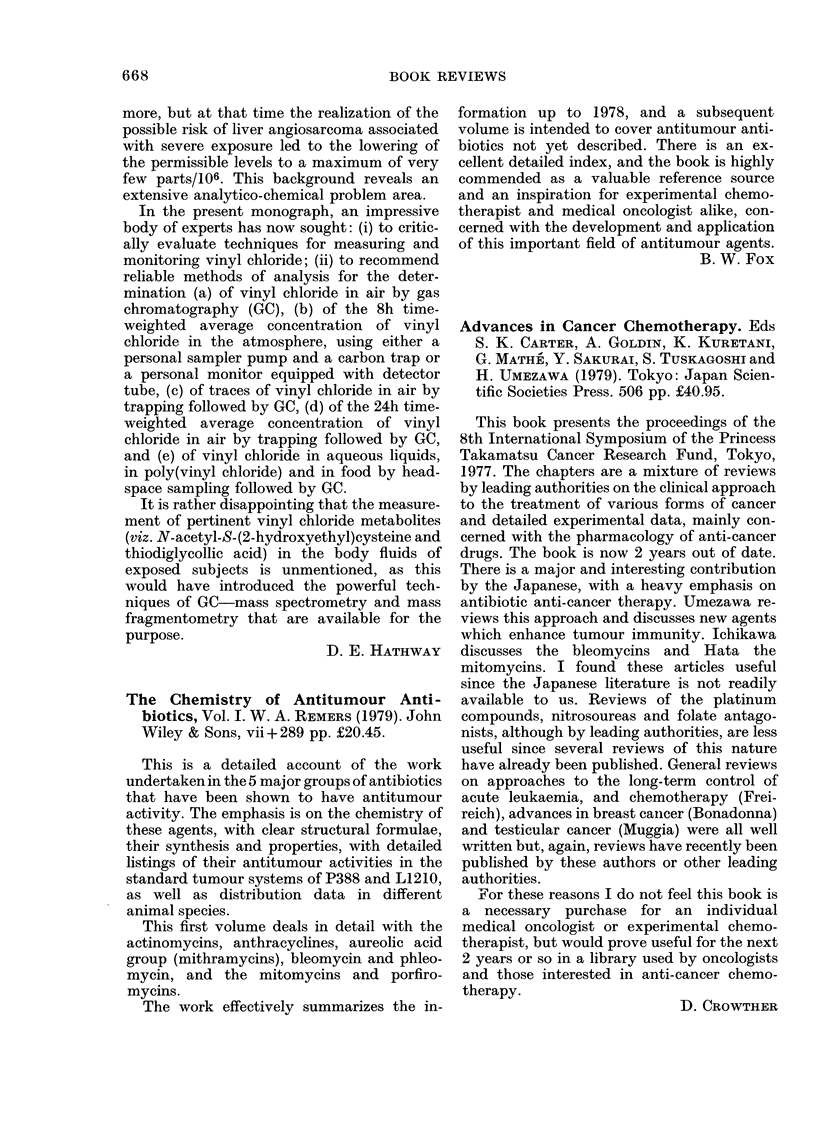# Environmental Carcinogens Selected Methods of Analysis, Vol. 2: Methods for Measurement of Vinyl Chloride in Poly(Vinyl Chloride), Air, Water and Foodstuffs

**Published:** 1979-10

**Authors:** D. E. Hathway


**Environmental Carcinogens Selected Methods of Analysis,** Vol. 2: **Methods for Measurement of Vinyl Chloride in Poly(Vinyl Chloride), Air, Water and Foodstuffs.** D. C. M. SQUIRRELL, W. THAIN (Ed. H. EGAN) (1978). IARC Scientific Publications No. 22. 140 pp. Sw.fr.75.

In 1974, a working group (of a few of us who were involved) was convened by the Agency to attempt to evaluate the risk of vinyl chloride carcinogenicity to man. Before then, the permissible level of vinyl chloride exposure was of the order of 100 parts/$10^6$ or more, but at that time the realization of the possible risk of liver angiosarcoma associated with severe exposure led to the lowering of the permissible levels to a maximum of very few parts/$10^6$. This background reveals an extensive analytico-chemical problem area.

In the present monograph, an impressive body of experts has now sought: (i) to critically evaluate techniques for measuring and monitoring vinyl chloride; (ii) to recommend reliable methods of analysis for the determination (a) of vinyl chloride in air by gas chromatography (GC), (b) of the 8h time-weighted average concentration of vinyl chloride in the atmosphere, using either a personal sampler pump and a carbon trap or a personal monitor equipped with detector tube, (c) of traces of vinyl chloride in air by trapping followed by GC, (d) of the 24h time-weighted average concentration of vinyl chloride in air by trapping followed by GC, and (e) of vinyl chloride in aqueous liquids, in poly(vinyl chloride) and in food by head-space sampling followed by GC.

It is rather disappointing that the measurement of pertinent vinyl chloride metabolites (*viz. N*-acetyl-*S*-(2-hydroxyethyl)cysteine and thiodiglycollic acid) in the body fluids of exposed subjects is unmentioned, as this would have introduced the powerful techniques of GC—mass spectrometry and mass fragmentometry that are available for the purpose.

D. E. Hathway